# Variability of body mass index and risks of prostate, lung, colon, and ovarian cancers

**DOI:** 10.3389/fpubh.2022.937877

**Published:** 2022-08-25

**Authors:** Yangyang Sun, Lingling Zhou, Tao Shan, Qiong Ouyang, Xu Li, Yuanming Fan, Ying Li, Hang Gong, Raphael N. Alolga, Gaoxiang Ma, Yuqiu Ge, Heng Zhang

**Affiliations:** ^1^Department of Pharmacy, The Children's Hospital, Zhejiang University School of Medicine, National Clinical Research Center for Child Health, Hangzhou, China; ^2^State Key Laboratory of Natural Medicines, School of Traditional Chinese Pharmacy, China Pharmaceutical University, Nanjing, China; ^3^Clinical Metabolomics Center, China Pharmaceutical University, Nanjing, China; ^4^Department of Orthopaedic Surgery, Children's Hospital of Nanjing Medical University, Nanjing, China; ^5^Department of Anesthesiology, Nanjing First Hospital, Nanjing Medical University, Nanjing, China; ^6^Department of Pharmacy, JiangXi PingXiang People's Hospital, Pingxiang, China; ^7^Department of Public Health and Preventive Medicine, Wuxi School of Medicine, Jiangnan University, Wuxi, China; ^8^Department of Hematology and Oncology, Children's Hospital of Nanjing Medical University, Nanjing, China

**Keywords:** BMI, variability, lung cancer, PLCO cohort study, risk factor

## Abstract

**Objective:**

We investigated the association between cancer incidence and body mass index (BMI) variability calculated from the recall of weight at decades of age by participants in the USA Prostate, Lung, Colorectal and Ovarian (PLCO) Cancer Screening Trial.

**Methods:**

A total of 89,822 individuals' BMI were recorded as recalled the participant's aged 30, 40, 50, 60, 70 years, and baseline. BMI variability was assessed using four indices: SD, coefficient of variation (CV), variability independent of the mean (VIM), and average real variability (ARV). The multivariate Cox regression analysis was performed to calculate hazard ratios (HRs) of these measures for incident cancers and corresponding 95% CIs.

**Results:**

During the median follow-up of 11.8 years, there were newly diagnosed 5,012 cases of prostate cancer, 792 cases of lung cancer, 994 cases of colon cancer, and 132 cases of ovarian cancer. Compared with the lowest quartile (Q1) group, the highest quartile (Q4) group of BMI variability indices was associated with increased lung cancer risk, including BMI_SD (HR, 1.58; 95% CI, 1.17–2.12), BMI_CV (HR, 1.46; 95% CI, 1.10–1.94), BMI_VIM (HR, 1.73; 95% CI, 1.33–2.25), and BMI_ARV (HR, 2.17; 95% CI, 1.62–2.91). Associations between BMI variability and prostate, colon, and ovarian cancer incidences were of limited significance.

**Conclusion:**

The findings imply that maintaining a stable weight across adulthood is associated with a decreased incidence of lung cancer.

## Introduction

Cancer Statistics reported 19.3 million new cancer cases and almost 10.0 million cancer deaths globally in 2020 (1), including ~1.8 million new cancer cases and 0.6 million cancer deaths in the USA (2). Thereby, cancer remained one of the leading causes of death. There is an urgent need to identify and avoid exposure to potential risk factors by instituting appropriate interventions (3).

Obesity is an independent risk factor for cancer (4). Increasing evidence has revealed that body mass index (BMI) [weight (kg)/height (m^2^)], the most common indicator of obesity, was associated with the incidence of multiple cancer (5–7). The performance of BMI measurement at a specific time is not able to represent long-term weight changes. The variability of BMI could depict weight changes; thus, it is meaningful to assess the association between the variability of BMI and cancer risks.

Existing studies indicated that the variability of BMI may affect the incidence of adverse outcomes (8, 9). Body weight variability increased the risk of dementia and cardiovascular diseases and mortality of patients with type 2 diabetes based on a nationwide population-based cohort from Korea (10, 11). In addition, BMI variability was reported to increase the risk of atrial fibrillation, myocardial infarction, and all-cause mortality (12, 13). But no associations between BMI variability and risk of breast, endometrial, colon, or lung cancers were found in the Iowa Women's Health Study (14). To this end, the association between BMI variability and cancer risk remains largely obscure.

To address the gaps in current knowledge, this study evaluated the relationship of BMI variability from adulthood to elderhood and the incidence of prostate, lung, colon, and ovarian cancers using the Prostate, Lung, Colorectal and Ovarian (PLCO) Cancer Screening Trial.

## Materials and methods

### Study population

The Prostate, Lung, Colorectal and Ovarian Cancer Screening Trial was designed to intervene the prostate, lung, colon, and ovarian cancers mortality by screening methods (https://cdas.cancer.gov/plco/). The overall design of the PLCO has been described elsewhere (15–17). From November 1993 to July 2001, ~1,550,000 participants aged 55–74 years were randomly assigned to the control or intervention groups at 10 screening centers in the United States. We followed the reporting guidelines of the Strengthening the Reporting of Observational Studies in Epidemiology (STROBE) statement for observational studies.

### Anthropometric measurements and index of body mass index variability

All the participants were asked, “Please estimate your weight and height when you were in your 30, 40, 50, 60, 70s, and current,” respectively. BMI was calculated as an individual's weight in kilograms divided by the square of their height in meters. To comprehensively investigate the association between BMI variability and the risk of cancers, BMI variability was assessed using four indices: (1) SD; (2) coefficient of variation (CV); (3) variability independent of the mean (VIM); and (4) average real variability (ARV). VIM was calculated as 100 × SD/mean^β^, where β is the regression coefficient based on the ln of the SD over the ln of the mean (18). ARV is the average of the absolute differences between consecutive values and was calculated using the following formula: ARV = $\frac{1}{n-1}\overset{n-1}{\underset{k=1}{\sum }}|Value_{k+1}-Value_{k}|$, where *n* denotes the number of anthropometric measurements (19). The earliest recorded BMI greater than the latest recorded BMI was regarded as BMI loss. On the contrary, a lower earliest recorded BMI than the latest value was defined as BMI gain.

### Study outcome

Newly diagnosed prostate, lung, colon, and ovarian cancers were treated as the endpoints of this study. The Annual Study Update (ASU) was used to ascertain cancer diagnosis. Participants were asked if they were diagnosed with cancer, the type of cancer, date of diagnosis, hospital or clinic of diagnosis, and physician contact information. Prostate cancer (C61.9), lung cancer (C34.1 to C34.9), colon cancer (C18.0 to C18.9), and ovarian cancer (C56.9) sites had the International Classification of Disease for Oncology, third edition (ICD-O-3) codes based on initial medical records.

### Definition of covariates

Self-reporting questionnaires were used to obtain demographic and lifestyle data. The baseline questionnaire (BQ) and supplemental questionnaire (SQ) included information about demographics, history of health, smoking, drugs used, and gender-specific details. The Diet History Questionnaire (DHQ) is a food frequency questionnaire. Educational level was defined as <8, 8–11, 12 years, or completed high school, posthigh school training other than college, some college, college graduate, and postgraduate. Smoking status was categorized as non-smoker, former smoker, and current smoker. Drinking status was dichotomized into never, former, and current drinker. The race was classified as white (non-Hispanic), black (non-Hispanic), Hispanic, Asian, Pacific Islander, and American-Indian. Physical activity was defined as whether participants exercised 1+ time/month. Dietary covariates (drinking statuses, vegetable and fruit consumption, and vitamin D intake) contained in the Diet History Questionnaire, which was a food frequency questionnaire that was added in 1998, not baseline data. Other variables recorded baseline questionnaire was baseline data.

### Exclusion criteria

**Figure 1:**
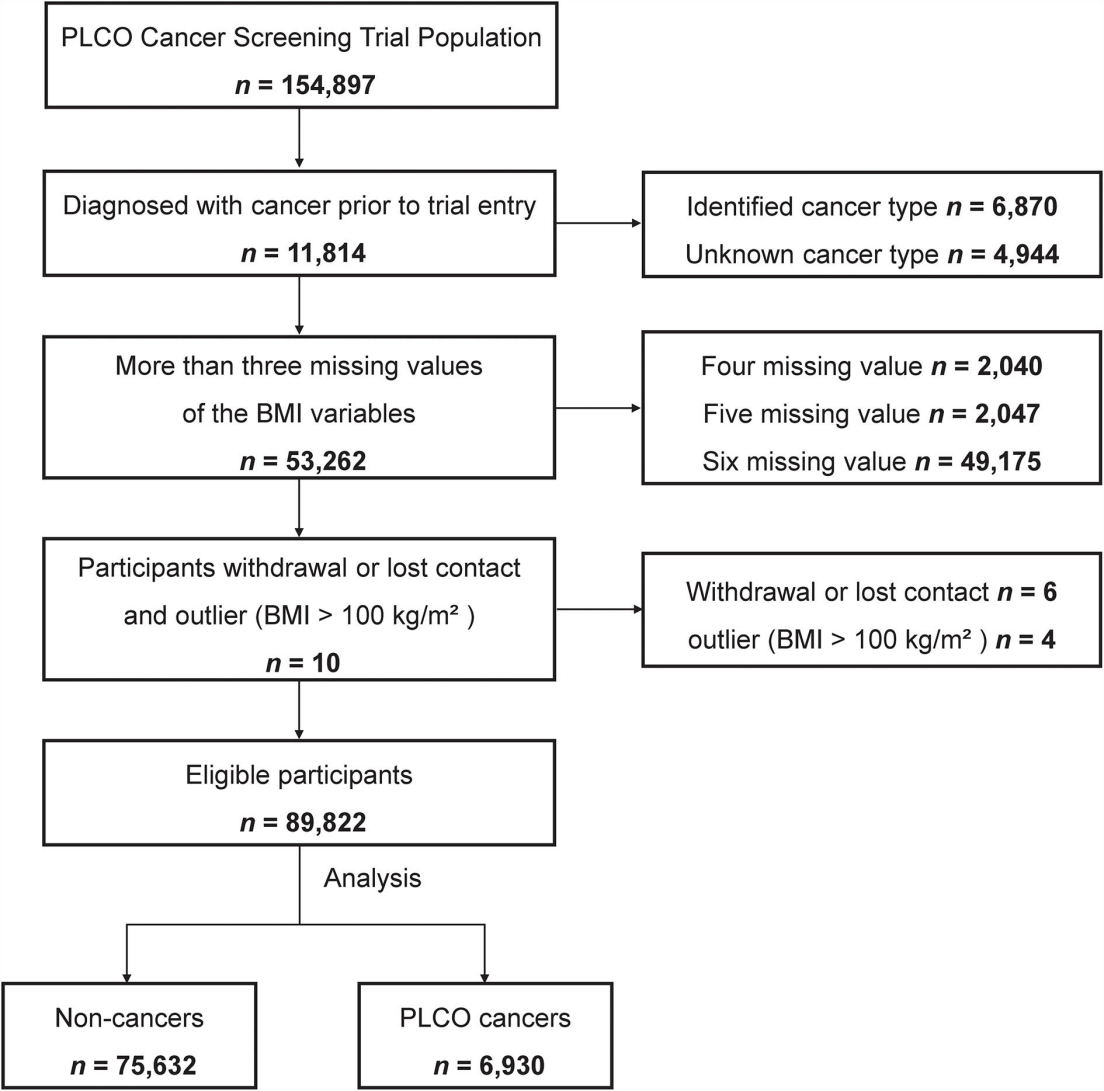
Flowchart of inclusion criteria.

Pertinent exclusion criteria for the current analysis were as follows: (1) diagnosis with any cancer prior to trial entry (*n* = 11,814); (2) more than three missing values of the BMI variables (*n* = 53,262); (3) participants withdrawal or lost contact (*n* = 6); and (4) BMI > 100 kg/m^2^ (*n* = 4). Finally, 89,822 individuals with three or more available body weight and height measurements were enrolled in this study. The study participants were followed-up until 31 December 2009. The mean follow-up duration was 11.0 ± 2.7 years. The flowchart of the study population is shown in Figure 1.

### Statistical analysis

The baseline characteristics of the study participants are presented as mean ± SD for continuous variables and number (percentage) for categorical variables. The incidence rates of outcomes (prostate, lung, colon, and ovarian cancers) were calculated by dividing the number of events by 1,000 person-years. The multivariate Cox regression models were performed to examine the associations between quartiles of BMI variability and the risk of outcomes. Hazard ratios (HRs) and corresponding 95% CI were calculated compared with the lowest quartile group. The repeated measures Cox regression was used to investigate the association between repeated BMI measurements and cancers.

We also conducted three sensitivity analyses to determine the robustness of our findings. First, we calculated four indices of BMI variability for roundly representing variability. Second, the participants diagnosed with other cancers (cancer was not the outcome event of concern) were censored during follow-up before the onset of outcomes in our study. It is necessary to examine the possible influence of competing events on the association between BMI variability and the risk of lung cancer by applying Fine and Gray's subdistribution hazards regression model (20). Finally, considering possible reverse causality, we reanalyzed the association between quartiles of BMI variability and the risk of study outcomes by excluding participants whose outcomes event occurred in the first 2 years of follow-up.

We performed subgroup analyses of age, sex, smoking status, BMI trajectory, and baseline BMI. We evaluated the associations between BMI variability and the risk of cancer in these subgroups. To quantify dose-response relationships, we used restricted cubic spline models with four knots at the 5, 35, 65, and 95th centiles to examine the associations between BMI variability indices (SD, ARV, CV, and VIM as continuous variables) and the risk of cancer after full adjustment. All the statistical tests were two-sided, and *P* <0.05 was considered statistically significant. Analyses were performed using R software (version 3.6.1).

### Ethics approval and consent to participate

All the methods and experiments were approved by the China Pharmaceutical University Ethics Committee. All the participants provided written informed consent. This study was carried out in accordance with the Declaration of Helsinki.

## Results

### Baseline characteristics

The average age at baseline of the eligible PLCO study population of the 89,822 participants was 62.0 ± 5.1 years. As shown in Table 1, there were 43,510 (48.4%) males, 82,356 (91.7%) white, no Hispanic, and 7,652 (8.5%) current smokers. The outcomes of current study included prostate cancer cases (*n* = 5,012), lung cancer cases (*n* = 792), colon cancer cases (*n* = 994), and ovarian cancer cases (*n* = 132).

**Table 1:** Baseline characteristics of the study population.

**Characteristic**	**Cohort**	**Prostate cancer**	**Lung cancer**	**Colon cancer**	**Ovarian cancer**
No. of participants	89,822	5,012	792	994	132
Age, mean (SD), years	62.0 (5.1)	63.0 (5.0)	62.8 (5.1)	63.6 (5.1)	62.9 (5.1)
Sex, men (%)	43,510 (48.4)	5,012 (100.0)	414 (52.3)	547 (55.0)	0 (0.0)
White, Non-Hispanic (%)	82,356 (91.7)	4,612 (92.0)	732 (92.4)	906 (91.1)	122 (92.4)
Current smoker (%)	7,652 (8.5)	364 (7.3)	321 (40.5)	108 (10.9)	9 (6.8)
Current drinker (%)	58,925 (74.2)	3,418 (77.0)	524 (77.4)	646 (73.7)	94 (77.0)
Height (%), inches	67.1 (3.9)	70.1 (2.7)	67.5 (4.0)	67.6 (4.1)	64.6 (2.9)
Weight (%), lbs	174 (36.4)	190 (29.4)	172 (35.5)	180 (37.3)	158 (35.7)
Baseline BMI, mean (SD), kg/m^2^	27.2 (4.7)	27.2 (3.8)	26.4 (4.4)	27.7 (4.8)	26.8 (5.8)
BMI in 30s, mean (SD), kg/m^2^	23.3 (3.4)	24.1 (3.2)	23.1 (3.4)	23.6 (3.5)	22.0 (3.2)
BMI in 40s, mean (SD), kg/m^2^	24.4 (3.7)	25.0 (3.2)	23.9 (3.6)	24.7 (3.6)	23.2 (4.0)
BMI in 50s, mean (SD), kg/m^2^	25.8 (4.2)	26.0 (3.5)	25.2 (3.8)	26.1 (4.1)	24.6 (4.2)
BMI in 60s, mean (SD), kg/m^2^	27.0 (4.7)	27.1 (3.9)	26.3 (4.2)	27.3 (4.9)	26.3 (5.4)
BMI in 70s, mean (SD), kg/m^2^	26.8 (4.6)	27.2 (3.9)	26.1 (4.3)	27.4 (4.8)	26.3 (4.8)
Current BMI, mean (SD), kg/m^2^	27.4 (5.0)	27.5 (4.1)	26.4 (4.7)	27.7 (5.2)	26.6 (5.5)
BMI_SD, mean (SD)	2.3 (1.7)	1.9 (1.4)	2.2 (1.5)	2.4 (1.7)	2.5 (1.8)
BMI_CV, mean (SD)	8.5 (6.0)	7.3 (4.8)	8.7 (5.4)	8.7 (5.4)	9.4 (6.0)
BMI_VIM, mean (SD)	1.5e-4 (0.9e-4)	1.5e-4 (0.9e-4)	1.6e-4 (1.0e-4)	1.7e-4 (0.9e-4)	1.3e-4 (0.8e-4)
BMI_ARV, mean (SD)	1.4 (1.2)	1.2 (0.9)	1.4 (1.0)	1.5 (1.1)	1.7 (1.5)
Family history (%)
Prostate cancer	8,882 (11.1)	889 (19.8)	65 (9.27)	88 (10.1)	17 (14.7)
Lung cancer	11,265 (14.0)	579 (13.0)	172 (24.4)	140 (16.0)	23 (20.0)
Colon cancer	9,661 (12.0)	487 (10.9)	86 (12.2)	152 (17.4)	11 (9.6)
Ovarian cancer	4,374 (5.5)	210 (4.7)	40 (5.7)	52 (6.0)	10 (8.7)

### Associations between body mass index variability, repeated body mass index measurements, and risk of study outcomes

Table 2 presents the relationship between BMI variability indices (BMI_SD and BMI_ ARV) and prostate, lung, colon, and ovarian cancer risks. After adjusting for age, sex, and baseline BMI (model 1), individuals with the highest quartile (Q4) of BMI_SD and BMI_ARV were at significantly higher risk of lung cancer (HR, 95% CI = 1.76, 1.38–2.25, *P*_trend_ <0.001 for BMI_SD; 2.18, 1.71–2.77, *P*_trend_ <0.001 for BMI_ARV) compared with those with the lowest quartile (Q1). After adjusting for all the potential confounding variables (model 2), individuals with the highest quartile of BMI_SD and BMI_ARV had a higher risk of lung cancer (HR, 95% CI = 1.58, 1.17–2.12, *P*_trend_ = 0.001 for BMI_SD; 2.17, 1.62–2.91, *P*_trend_ <0.001 for BMI_ARV) compared with those with the lowest quartile (Table 2). These associations were also observed for BMI_CV and BMI_VIM (Supplementary Table 1).

**Table 2:** SD and ARV for BMI variability in relation to the risks of prostate, lung, colon, and ovarian cancers.

					**Without competing risks**		**With competing risks**	
**Outcomes**	***N***	**Cases**	**PYs**	**IR^a^**	**Model 1**	**Model 2**	**Model 1**	**Model 2**
**Lung cancer**		792						
**SD**								
Q1	22,439	177	249,141	0.71	1.00 (reference)	1.00 (reference)	1.00 (reference)	1.00 (reference)
Q2	22,452	209	247,395	0.84	1.33 (1.09–1.64)	1.33 (1.03–1.70)	1.32 (1.08–1.62)	1.31 (1.02–1.69)
Q3	22,475	215	246,546	0.87	1.56 (1.26–1.93)	1.51 (1.17–1.95)	1.54 (1.24–1.91)	1.49 (1.15–1.93)
Q4	22,456	191	244,408	0.78	1.76 (1.38–2.25)	1.58 (1.17–2.12)	1.73 (1.34–2.24)	1.55 (1.14–2.12)
*P*_trend_					**<0.001**	**0.001**	**<0.001**	**0.003**
**ARV**								
Q1	22,559	164	250,951	0.65	1.00 (reference)	1.00 (reference)	1.00 (reference)	1.00 (reference)
Q2	22,419	213	247,379	0.86	1.52 (1.23–1.87)	1.56 (1.20–2.01)	1.51 (1.22–1.86)	1.54 (1.19–1.99)
Q3	22,388	208	245,414	0.85	1.75 (1.41–2.18)	1.76 (1.35–2.29)	1.73 (1.39–2.15)	1.72 (1.32–2.26)
Q4	22,456	207	243,746	0.85	2.18 (1.71–2.77)	2.17 (1.62–2.91)	2.13 (1.66–2.73)	2.12 (1.57–2.86)
*P*_trend_					**<** **0.001**	**<** **0.001**	**<** **0.001**	**<** **0.001**
**Colon cancer**		994						
**SD**								
Q1	22,439	211	249,141	0.85	1.00 (reference)	1.00 (reference)	1.00 (reference)	1.00 (reference)
Q2	22,452	243	247,395	0.98	1.13 (0.93–1.36)	1.04 (0.84–1.30)	1.12 (0.93–1.36)	1.04 (0.83–1.30)
Q3	22,475	285	246,546	1.16	1.32 (1.09–1.60)	1.22 (0.98–1.53)	1.31 (1.08–1.58)	1.21 (0.97–1.52)
Q4	22,456	255	244,408	1.04	1.26 (1.01–1.57)	1.13 (0.87–1.47)	1.24 (1.00–1.54)	1.12 (0.86–1.45)
*P*_trend_					**0.01**	0.19	**0.02**	0.21
**ARV**								
Q1	22,559	206	250,951	0.82	1.00 (reference)	1.00 (reference)	1.00 (reference)	1.00 (reference)
Q2	22,419	241	247,379	0.97	1.22 (1.00–1.47)	1.19 (0.95–1.48)	1.21 (1.00–1.46)	1.18 (0.95–1.48)
Q3	22,388	286	245,414	1.17	1.46 (1.20–1.77)	1.37 (1.09–1.71)	1.45 (1.19–1.75)	1.35 (1.08–1.70)
Q4	22,456	261	243,746	1.07	1.42 (1.14–1.76)	1.29 (1.00–1.68)	1.39 (1.12–1.73)	1.27 (0.98–1.66)
*P*_trend_					**<0.001**	**0.03**	**<0.001**	**0.04**
**Prostate cancer**		5,012						
**SD**								
Q1	10,878	1,272	119,722	10.62	1.00 (reference)	1.00 (reference)	1.00 (reference)	1.00 (reference)
Q2	10,877	1,265	119,016	10.63	1.07 (0.99–1.15)	1.05 (0.96–1.14)	1.01 (0.93–1.09)	0.98 (0.89–1.07)
Q3	10,877	1,306	117,983	11.07	1.06 (0.98–1.15)	1.06 (0.96–1.16)	1.08 (1.00–1.17)	1.07 (0.98–1.18)
Q4	10,878	1,169	117,438	9.95	1.06 (0.96–1.16)	1.07 (0.95–1.19)	1.01 (0.93–1.11)	1.02 (0.92–1.14)
*P*_trend_					0.23	0.23	0.29	0.28
**ARV**								
Q1	10,883	1,306	119,961	10.89	1.00 (reference)	1.00 (reference)	1.00 (reference)	1.00 (reference)
Q2	10,880	1,307	119,047	10.98	1.02 (0.95–1.11)	1.03 (0.94–1.13)	1.02 (0.94–1.10)	1.02 (0.94–1.12)
Q3	10,861	1,272	117,952	10.78	1.03 (0.95–1.11)	1.03 (0.94–1.14)	1.02 (0.94–1.10)	1.03 (0.93–1.13)
Q4	10,886	1,127	117,199	9.62	0.96 (0.88–1.05)	0.98 (0.89–1.09)	0.95 (0.87–1.04)	0.97 (0.88–1.08)
*P*_trend_					0.47	0.87	0.51	0.77
**Ovarian cancer**		132						
**SD**								
Q1	11,577	39	129,780	0.30	1.00 (reference)	1.00 (reference)	1.00 (reference)	1.00 (reference)
Q2	11,578	30	129,042	0.23	0.81 (0.50–1.33)	0.72 (0.41–1.24)	0.81 (0.50–1.32)	0.72 (0.41–1.24)
Q3	11,581	30	127,870	0.23	0.85 (0.50–1.46)	0.69 (0.37–1.28)	0.85 (0.50–1.42)	0.68 (0.37–1.23)
Q4	11,576	33	126,638	0.26	0.99 (0.53–1.85)	0.97 (0.47–2.02)	0.97 (0.54–1.75)	0.96 (0.48–1.91)
*P*_trend_					0.91	0.70	0.75	0.74
**ARV**								
Q1	11,555	36	129,821	0.28	1.00 (reference)	1.00 (reference)	1.00 (reference)	1.00 (reference)
Q2	11,770	28	131,276	0.21	0.83 (0.50–1.38)	0.79 (0.45–1.40)	0.83 (0.50–1.38)	0.79 (0.44–1.41)
Q3	11,312	36	124,863	0.29	1.22 (0.73–2.05)	1.08 (0.59–1.98)	1.21 (0.73–1.98)	1.07 (0.60–1.90)
Q4	11,679	32	127,370	0.25	1.14 (0.61–2.11)	1.35 (0.67–2.73)	1.12 (0.64–1.95)	1.33 (0.71–2.47)
*P*_trend_					0.46	0.37	0.88	0.84

PYs, person-years; IR, incidence rate.

a Incidence per 1,000 person-years. Model 1 was adjusted for age, sex and BMI at baseline. Model 2 of lung cancer was adjusted for age, sex, education, BMI at baseline, race, smoking and drinking status, randomization arm, vegetable and fruit consumption, family history of lung cancer, vitamin D intake, physical activity and the cross-product term of physical activity and fruit intake. Model 2 of colon cancer was adjusted for age, sex, education, BMI at baseline, race, smoking and drinking status, randomization arm, vegetable and fruit consumption, family history of colon cancer, vitamin D intake, physical activity and colon comorbidities. Model 2 of prostate cancer was adjusted for age, education, BMI at baseline, race, smoking and drinking status, randomization arm, vegetable and fruit consumption, family history of prostate cancer, vitamin D intake and physical activity. Model 2 of ovarian cancer was adjusted for age, education, BMI at baseline, race, smoking and drinking status, randomization arm, vegetable and fruit consumption, family history of ovarian cancer, vitamin D intake and physical activity. The bold values mean ‘P <0.05'.

In addition, the association between BMI variability and the risk of colon cancer was significantly increased in the higher quartile groups of SD and ARV in model 1 (HR, 1.29; 95% CI, 1.00–1.68, *P*_trend_ = 0.005 for BMI_SD; HR, 1.29; 95% CI, 1.00–1.68, *P*_trend_ = 0.005 for BMI_ARV, respectively) compared with the lowest quartile (Q1) group (Table 2). The association between ARV and the risk of colon cancer was significantly increased in the fully adjusted models (HR, 1.29; 95% CI, 1.00–1.68, *P*_trend_ = 0.026) compared with the lowest quartile (Q1) group (Table 2). We did not observe any association between BMI_SD, BMI_CV, and BMI_VIM and the risk of colon cancer (Table 2; Supplementary Table 1). Similarly, no significant relationship between BMI variability and prostate and ovarian cancer risks was observed in model 1 or model 2 (data not shown). The association between repeated BMI measurements and risk of cancers was not observed based on the repeat measurement Cox model (Supplementary Table 3).

**Figure 2:**
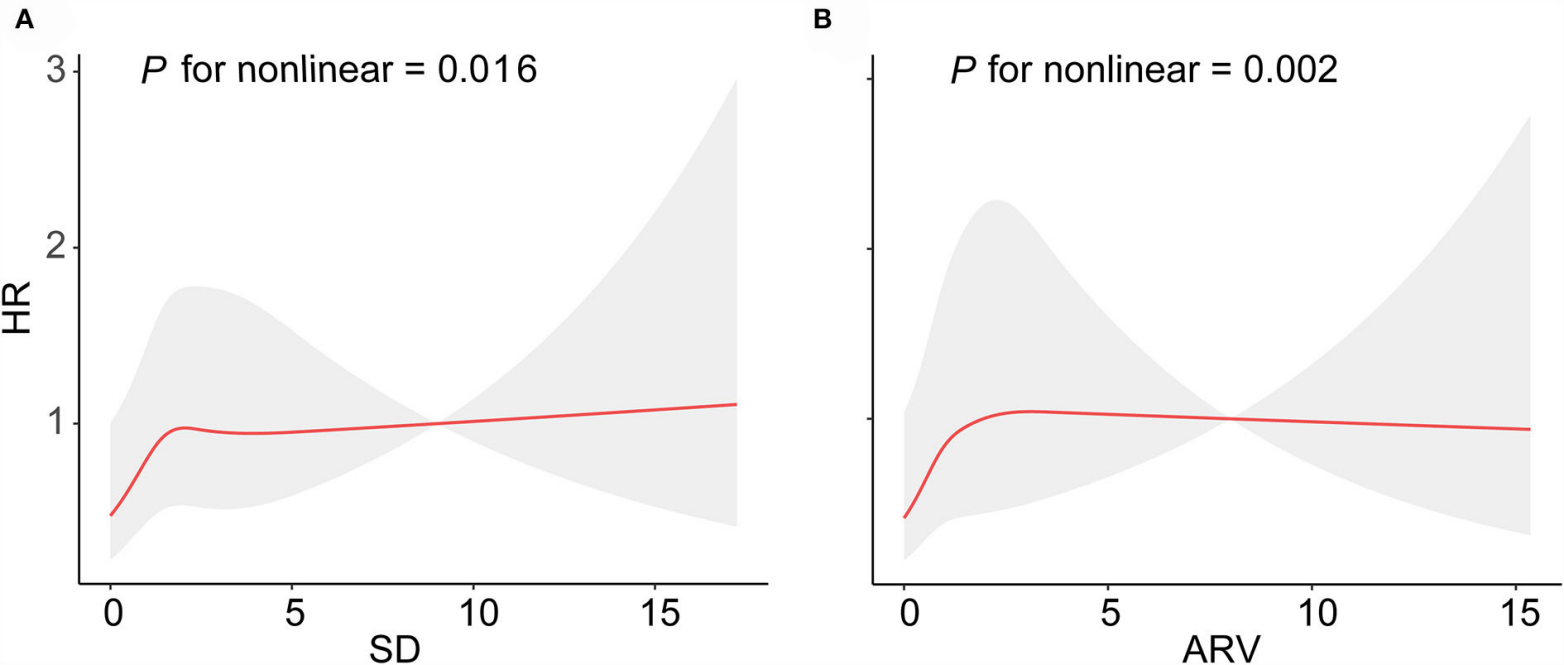
Curve association between SD and ARV for BMI variability and the risk of lung cancer. Shading indicates 95% CIs. **(A)** SD for BMI, **(B)** CV for BMI.

We used restricted cubic splines to flexibly model and visualize the relationship of BMI variability with the risk of lung cancer. The curves for the associations between BMI_SD (Figure 2A) and BMI_ARV (Figure 2B) and the risk of lung cancer were non-linear (*P* for non-linear = 0.016, *P* for non-linear = 0.002, respectively). For BMI_ARV, the risk curve displayed an inverse U-shape (Figure 2B). As shown in Supplementary Figure 2, the BMI_CV and BMI_VIM were linearly associated with incident lung cancer (*P* for non-linear = 0.098, *P* for non-linear = 0.404, respectively).

### Subgroup analysis

**Figure 3:**
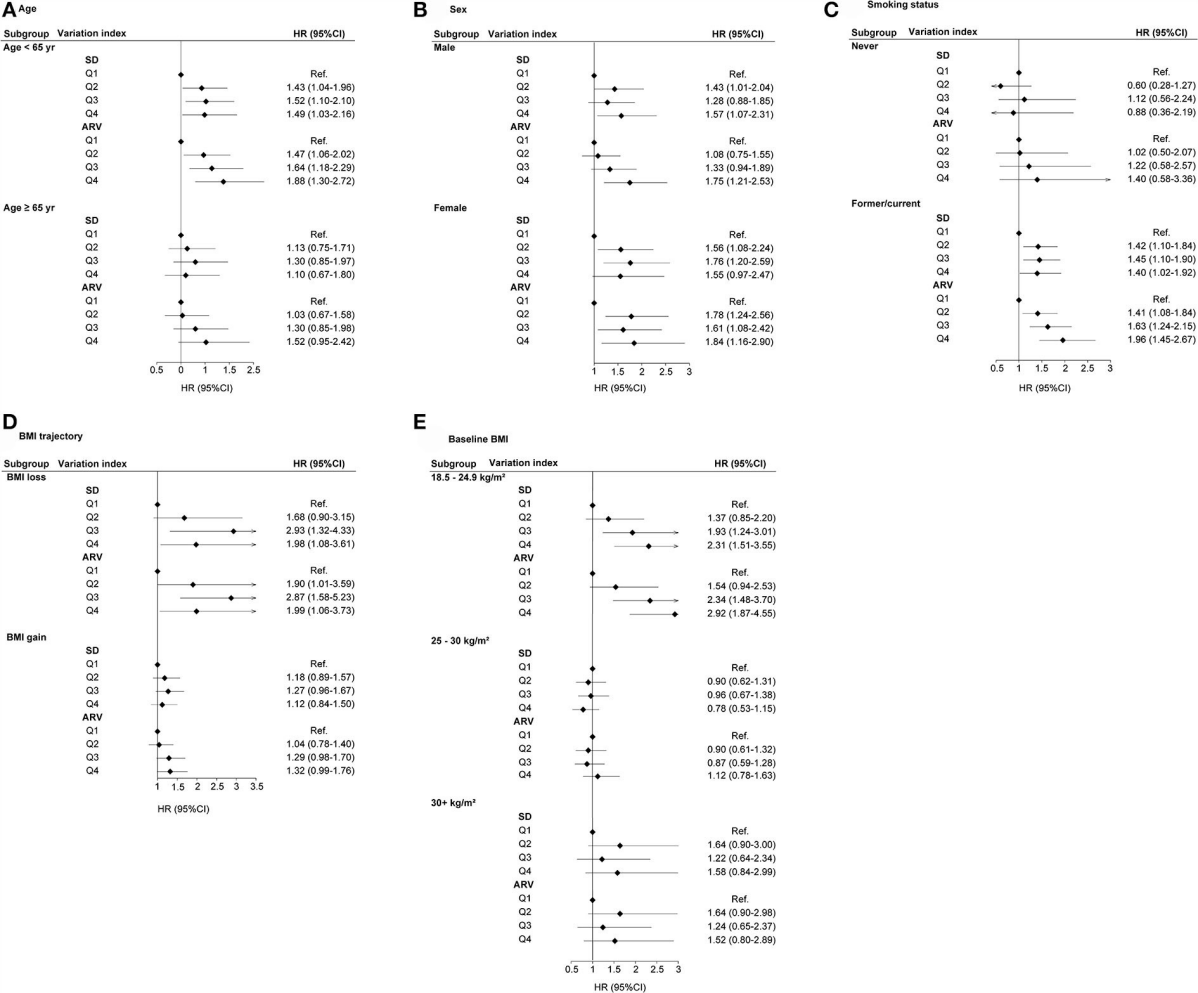
HR (95% CI) of lung cancer with respect to SD and ARV for BMI variability in subgroups. **(A)** Age, **(B)** Sex, **(C)** Smoking status, **(D)** BMI trajectory, and **(E)** Baseline BMI.

The subgroup analysis was performed for the risk of lung cancer based on the Q1–Q4 groups of BMI_SD, BMI_CV, BMI_VIM, and BMI_ARV. BMI variability (SD, ARV, CV, and VIM) was associated with higher risks of lung cancer at age <65 years (Figure 3A; Supplementary Figure 1A). For sex, higher BMI variability was consistently associated with incident lung cancer, except for BMI_CV in males (Figure 3B; Supplementary Figure 1B), and the positive association between BMI variability and lung cancer was limited to former and current smokers (Figure 3C; Supplementary Figure 1C). BMI variability was associated with higher risks of lung cancer in BMI loss, except for BMI_VIM (Figure 3D; Supplementary Figure 1D). BMI variability was associated with higher risks of lung cancer with a baseline BMI of 18.5–24.9 kg/m^2^, except for BMI_VIM (Figure 3E; Supplementary Figure 1E).

### Sensitivity analysis

The four indices of BMI variability (SD, ARV, CV, and VIM) also demonstrated a consistent association with risks of the PLCO cancer (Table 2; Supplementary Table 1). Then, we considered the incident non-PLCO cancer as competing events, the results of which revealed similar association patterns between BMI variability and risk of the PLCO cancers (Table 2; Supplementary Table 1). Last, when we excluded the PLCO cancer cases that had been ascertained in the first 2 years of follow-up, an association between BMI variability (SD, ARV, CV, and VIM) and risks of the PLCO cancers did not change substantially (Supplementary Table 2). Yet after adjusting for potential confounding variables, SD and CV of BMI were also positively related to the incidence of colon cancer (Q3: HR, 1.33; 95% CI, 1.02–1.72, *P*_trend_ = 0.032 for BMI_SD, Q3: HR, 1.38; 95% CI, 1.07–1.79, *P*_trend_ = 0.041 for BMI_CV, respectively) compared to the lowest quartile (Q1) group (Supplementary Table 2). Individuals in the second quartile (Q2) and highest quartile (Q4) groups of BMI_VIM had a higher risk of colon cancer (Q2: HR, 1.35; 95% CI, 1.05–1.72, Q4: HR, 1.37; 95% CI, 1.05–1.79, *P*_trend_ = 0.089) compared to those in the lowest quartile (Q1) group (Supplementary Table 2).

## Discussion

Instability in BMI after 30 years of age was found to be associated with an increased risk of lung cancer after adjusting for potential risk factors in this study. Our findings suggested that higher BMI variability may have an effect on the incidence of lung cancer. Maintaining proper weight may be beneficial for lowering the risk of lung cancer.

To the best of our knowledge, only the Iowa Women's Health Study has examined the association between body weight (not BMI) variability and incident lung and colon cancers. They found that the root mean square error around the slope of weight on age defined as body weight variability was associated with an increased risk of lung cancer in model 1, but the association was attenuated after full adjustment (14). They found no association between body weight variability and the risk of colon cancer, which is similar to our study. Our study has some strengths compared to the previous study. The Iowa Women's Health study only reported the association between the risk of lung cancer and BMI variability in the women population. We found that the association between BMI variability and lung cancer was not limited to women. The follow-up duration was 2–6 years in the Iowa Women's Health study, while the participants' median follow-up duration was 11 years in our study. Besides, we had a bigger sample size of 89,822 subjects compared to 33,834 subjects for that study (14).

The underlying reasons for the association between increased BMI variability and the risk of lung cancer remain unclear. Previous observational studies demonstrated that weight loss was associated with loss of lean muscle mass, and weight regain was related to increased adiposity (21–23). This may lead to an increase in fat mass after body weight variabilities, resulting in an increase in adipose tissues. Adipose tissue secretes numerous adipocytokines that potentially played a role in the pathogenesis of lung cancer (22). For instance, stimulation of SQ-5 human clonal squamous lung cancer-derived cell proliferation was found after the addition of recombinant human leptin (24). Leptin seemed to mediate and amplify a complex interplay between tumor and immunoinflammatory cells, resulting in the development and progression of lung cancer (25). Moreover, the expression of adiponectin receptors exclusively in lung cancer tissues suggested that adiponectin functional signaling mediated lung cancer development (26). Further studies were warranted to explore the exact mechanism underlying the association between increased BMI variability and the risk of lung cancer.

We also found interesting results. The associations between BMI_SD, BMI_CV, and BMI_VIM and incident colon cancer were not observed after full adjustment, except for BMI_ARV. However, individuals in the third quartile (Q3) of BMI_SD and BMI_CV and in the second quartile (Q2) and highest quartile (Q4) of BMI_VIM had an increased risk of incident colon cancer when excluding colon cancer cases ascertained in the first 2 years of follow-up. This result of the sensitivity analysis contradicted the above results. It showed that the association between BMI variability and incident colon cancer needed to be further analyzed using another large sample size.

In subgroup analysis, the positive association of BMI variability with incident lung cancer existed in former or current smokers. This suggested that BMI variability was also a key risk factor for lung cancer in smokers. Furthermore, BMI variability was positively associated with incident lung cancer limited to individuals aged <65 years. This shows that it may be more important for maintaining weight before 65 years. These associations needed to be examined through further studies.

Our study had several notable strengths. We used a prospective study design, a large sample size of 89,822 individuals, and a follow-up period of >10 years. BMI of subjects in their 30, 40, 50, 60, 70s, and current age was provided for measuring long-term variability. Various subgroup analyses provided interesting conclusions. The stability of the results was confirmed by sensitivity analyses. Of note, this was the first study to investigate the association between BMI variability since adulthood and incident lung cancer during elderhood.

Several limitations deserve mention. First, a potential recall bias exists since the data on weight and height were self-reported. The authenticity of the data needs verification. Second, we could not determine whether the weight loss was intentional or unintentional due to a lack of information on the same. Third, we could not examine the association between BMI variability and the risk of non-PLCO cancers, since non-PLCO cancers had a low incidence in the PLCO cohort. Fourth, considering that the PLCO cohort was a controlled trial to determine whether certain screening examinations reduce mortality from prostate, lung, colorectal, and ovarian cancer. Participants for the intervention arm undergo a chest X-ray at baseline and annually for 2 years. In particular, participants classified as “smokers” undergo an additional chest X-ray at 3 years for lung screening. Lung screening and lifestyle interventions reduced the incidence of lung cancer and influenced participants' smoking behavior. Thereby, there is a low percentage of active smoking in the cohort, leading to a lower incidence of lung cancer in the cohort. Last, there is no denying that there are residual confounding factors in the association between BMI and cancers. Dietary carbohydrates and fiber from fruits, vegetables, and whole grains are associated with lower lung cancer risk. Refined carbohydrates, such as soft drinks, appear to increase risk based on the PLCO trial (27). In addition to dietary intake, a history of metabolic diseases associated with obesity, such as diabetes, has a significantly higher risk of lung cancer (28).

## Conclusion

This population-based study revealed that BMI variability is independently associated with an increased risk of lung cancer. Our findings suggested that maintaining a stable weight through appropriate interventions may be beneficial to preventing incident lung cancer, especially for smokers.

## Data availability statement

The datasets presented in this study can be found in online repositories. The names of the repository/repositories and accession number(s) can be found below: https://cdas.cancer.gov/plco/. This work has been conducted using the Prostate, Lung, Colorectal and Ovarian Cancer Screening Trial under project ID PLCO-411. Further information is available from the corresponding author upon request.

## Ethics statement

The studies involving human participants were reviewed and approved by China Pharmaceutical University Ethics Committee. The patients/participants provided their written informed consent to participate in this study.

## Author contributions

HZ and YG conceived and designed the study. YS, TS, and LZ conducted data analyses and prepared the first draft. All authors provided statistical expertise, aided in interpreting the results, contributed to the critical revision of the manuscript for important intellectual content, and approved the final version of the manuscript.

## Funding

This study was supported by the National Natural Science Foundation of China (81903390), the Jiangsu Provincial Natural Science Foundation (BK20190555 and BK20190600), the Science and Technology Development Foundation of Nanjing Medical University (NMUB20210063), and the Young Talent Support Project of Children's Hospital of Nanjing Medical University (TJGC2021014).

## Conflict of interest

The authors declare that the research was conducted in the absence of any commercial or financial relationships that could be construed as a potential conflict of interest.

## Publisher's note

All claims expressed in this article are solely those of the authors and do not necessarily represent those of their affiliated organizations, or those of the publisher, the editors and the reviewers. Any product that may be evaluated in this article, or claim that may be made by its manufacturer, is not guaranteed or endorsed by the publisher.
