# Supplementary material for: Variability of body mass index and risks of prostate, lung, colon, and ovarian cancers
Source: Front Public Health. 2022 Aug 25;10:937877. doi: 10.3389/fpubh.2022.937877 (PMC9452651; doi:10.3389/fpubh.2022.937877)
Supplement: Supplementary file 1 [file Data_Sheet_1.ZIP › Supplementary Material/Supplementary Figure legends.docx]

**Supplementary Figure legends**

**Supplementary Figure 1.** HR (95% CI) of the risk of lung cancer with respect to CV and VIM for BMI variability in subgroups. (A) Age, (B) Sex, (C) Smoking status, (D) BMI trajectory and (E) Baseline BMI.

**Supplementary Figure 2.** Curve association between CV and VIM for BMI variability and the risk of lung cancer. Shading indicates 95% CIs. (A) CV for BMI, (B) VIM for BMI.
