# Supplementary material for: Variability of body mass index and risks of prostate, lung, colon, and ovarian cancers
Source: Front Public Health. 2022 Aug 25;10:937877. doi: 10.3389/fpubh.2022.937877 (PMC9452651; doi:10.3389/fpubh.2022.937877)
Supplement: Supplementary file 1 [file Data_Sheet_1.ZIP › Supplementary Material/Supplementary Table.docx]

**Supplementary Table 1. CV and VIM for BMI variability in relation to the risk of prostate, lung, colon and ovarian cancers.**

|  |  |  |  |  | **Without competing risks** | | **With competing risks** | |
| --- | --- | --- | --- | --- | --- | --- | --- | --- |
|  | **N** | **Cases** | **Person-years** | **IR^*^** | **Model 1** | **Model 2** | **Model 1** | **Model 2** |
| **Lung cancer** |  | 792 |  |  |  |  |  |  |
| **CV** |  |  |  |  |  |  |  |  |
| Q1 | 22,456 | 172 | 249,005 | 0.69 | 1.00 (reference) | 1.00 (reference) | 1.00 (reference) | 1.00 (reference) |
| Q2 | 22,455 | 211 | 247,532 | 0.85 | 1.35 (1.10-1.66) | 1.34 (1.04-1.72) | 1.35 (1.09-1.65) | 1.32 (1.02-1.70) |
| Q3 | 22,455 | 213 | 246,291 | 0.86 | 1.53 (1.24-1.89) | 1.52 (1.18-1.96) | 1.52 (1.23-1.88) | 1.50 (1.16-1.94) |
| Q4 | 22,456 | 196 | 244,660 | 0.80 | 1.68 (1.33-2.12) | 1.46 (1.10-1.94) | 1.65 (1.30-2.10) | 1.43 (1.07-1.91) |
| *P*_trend_ |  |  |  |  | **<0.001** | **0.005** | **<0.001** | **0.006** |
| **VIM** |  |  |  |  |  |  |  |  |
| Q1 | 22,456 | 162 | 248,981 | 0.65 | 1.00 (reference) | 1.00 (reference) | 1.00 (reference) | 1.00 (reference) |
| Q2 | 22,455 | 204 | 246,903 | 0.83 | 1.39 (1.13-1.72) | 1.37 (1.06-1.78) | 1.38 (1.12-1.70) | 1.35 (1.04-1.75) |
| Q3 | 22,455 | 197 | 246,459 | 0.80 | 1.45 (1.17-1.80) | 1.40 (1.08-1.82) | 1.44 (1.16-1.79) | 1.38 (1.06-1.80) |
| Q4 | 22,456 | 229 | 245,146 | 0.93 | 1.89 (1.53-2.35) | 1.73 (1.33-2.25) | 1.86 (1.50-2.32) | 1.70 (1.30-2.21) |

**Supplementary Table 1. continued**

|  |  |  |  |  | **Without competing risks** | | **With competing risks** | |
| --- | --- | --- | --- | --- | --- | --- | --- | --- |
|  | **N** | **Cases** | **Person-years** | **IR^*^** | **Model 1** | **Model 2** | **Model 1** | **Model 2** |
| *P*_trend_ |  |  |  |  | **<0.001** | **<0.001** | **<0.001** | **<0.001** |
| **Colon cancer** |  | 994 |  |  |  |  |  |  |
| **CV** |  |  |  |  |  |  |  |  |
| Q1 | 22,456 | 213 | 249,005 | 0.86 | 1.00 (reference) | 1.00 (reference) | 1.00 (reference) | 1.00 (reference) |
| Q2 | 22,455 | 249 | 247,532 | 1.01 | 1.16 (0.96-1.39) | 1.13 (0.91-1.40) | 1.15 (0.96-1.39) | 1.12 (0.90-1.40) |
| Q3 | 22,455 | 282 | 246,291 | 1.14 | 1.31 (1.09-1.58) | 1.25 (1.00-1.56) | 1.30 (1.08-1.57) | 1.24 (1.00-1.55) |
| Q4 | 22,456 | 250 | 244,660 | 1.03 | 1.23 (1.00-1.52) | 1.13 (0.88-1.45) | 1.22 (0.99-1.50) | 1.12 (0.87-1.43) |
| *P*_trend_ |  |  |  |  | **0.023** | 0.22 | **0.028** | 0.25 |
| **VIM** |  |  |  |  |  |  |  |  |
| Q1 | 22,456 | 217 | 248,981 | 0.87 | 1.00 (reference) | 1.00 (reference) | 1.00 (reference) | 1.00 (reference) |
| Q2 | 22,455 | 271 | 246,903 | 1.10 | 1.27 (1.06-1.52) | 1.25 (1.01-1.54) | 1.26 (1.05-1.51) | 1.24 (1.00-1.54) |
| Q3 | 22,455 | 249 | 246,459 | 1.01 | 1.18 (0.98-1.43) | 1.10 (0.88-1.38) | 1.17 (0.97-1.42) | 1.10 (0.87-1.37) |
| Q4 | 22,456 | 257 | 245,146 | 1.05 | 1.27 (1.04-1.54) | 1.23 (0.97-1.55) | 1.25 (1.03-1.52) | 1.21 (0.96-1.53) |
| *P*_trend_ |  |  |  |  | **0.045** | 0.21 | 0.06 | 0.24 |

**Supplementary Table 1. continued**

|  |  |  |  |  | **Without competing risks** | | **With competing risks** | |
| --- | --- | --- | --- | --- | --- | --- | --- | --- |
|  | **N** | **Cases** | **Person-years** | **IR^*^** | **Model 1** | **Model 2** | **Model 1** | **Model 2** |
| **Prostate cancer** |  | 5,012 |  |  |  |  |  |  |
| **CV** |  |  |  |  |  |  |  |  |
| Q1 | 10,878 | 1,249 | 119,844 | 10.42 | 1.00 (reference) | 1.00 (reference) | 1.00 (reference) | 1.00 (reference) |
| Q2 | 10,877 | 1,264 | 118,969 | 10.62 | 1.03 (0.95-1.12) | 0.99 (0.91-1.09) | 1.02 (0.95-1.11) | 0.99 (0.90-1.08) |
| Q3 | 10,868 | 1,299 | 117,763 | 11.03 | 1.09 (1.01-1.18) | 1.06 (0.97-1.16) | 1.08 (1.00-1.17) | 1.05 (0.96-1.16) |
| Q4 | 10,877 | 1,200 | 117,583 | 10.21 | 1.04 (0.95-1.13) | 1.04 (0.94-1.15) | 1.03 (0.95-1.12) | 1.04 (0.94-1.15) |
| *P*_trend_ |  |  |  |  | 0.21 | 0.24 | 0.25 | 0.24 |
| **VIM** |  |  |  |  |  |  |  |  |
| Q1 | 10,878 | 1,241 | 119,695 | 10.37 | 1.00 (reference) | 1.00 (reference) | 1.00 (reference) | 1.00 (reference) |
| Q2 | 10,877 | 1,253 | 119,061 | 10.52 | 1.02 (0.94-1.10) | 0.99 (0.91-1.09) | 1.02 (0.94-1.10) | 0.99 (0.90-1.08) |
| Q3 | 10,882 | 1,263 | 118,252 | 10.68 | 1.05 (0.97-1.13) | 1.03 (0.94-1.13) | 1.04 (0.96-1.13) | 1.02 (0.93-1.12) |
| Q4 | 10,873 | 1,255 | 117,150 | 10.71 | 1.05 (0.97-1.14) | 1.05 (0.96-1.16) | 1.04 (0.96-1.13) | 1.05 (0.95-1.15) |
| *P*_trend_ |  |  |  |  | 0.197 | 0.22 | 0.150 | 0.110 |
| **Ovarian cancer** |  | 132 |  |  |  |  |  |  |

**Supplementary Table 1. continued**

|  |  |  |  |  | **Without competing risks** | | **With competing risks** | |
| --- | --- | --- | --- | --- | --- | --- | --- | --- |
|  | **N** | **Cases** | **Person-years** | **IR^*^** | **Model 1** | **Model 2** | **Model 1** | **Model 2** |
| **CV** |  |  |  |  |  |  |  |  |
| Q1 | 11,578 | 37 | 129,696 | 0.29 | 1.00 (reference) | 1.00 (reference) | 1.00 (reference) | 1.00 (reference) |
| Q2 | 11,579 | 30 | 128,921 | 0.23 | 0.86 (0.53-1.41) | 0.79 (0.45-1.38) | 0.86 (0.52-1.41) | 0.79 (0.45-1.38) |
| Q3 | 11,577 | 32 | 127,822 | 0.25 | 0..97 (0.58-1.62) | 0.92 (0.51-1.65) | 0.96 (0.58-1.59) | 0.91 (0.51-1.62) |
| Q4 | 11,577 | 33 | 126,878 | 0.26 | 1.05 (0.59-1.87) | 1.01 (0.51-2.00) | 1.04 (0.58-1.84) | 1.00 (0.52-1.92) |
| *P*_trend_ |  |  |  |  | 0.83 | 0.96 | 0.82 | 0.99 |
| **VIM** |  |  |  |  |  |  |  |  |
| Q1 | 11,578 | 33 | 129,567 | 0.25 | 1.00 (reference) | 1.00 (reference) | 1.00 (reference) | 1.00 (reference) |
| Q2 | 11,578 | 36 | 128,830 | 0.28 | 1.16 (0.72-1.87) | 1.15 (0.67-1.97) | 1.16 (0.72-1.86) | 1.15 (0.67-1.95) |
| Q3 | 11,578 | 29 | 128,186 | 0.23 | 0.97 (0.58-1.64) | 0.84 (0.46-1.56) | 0.97 (0.58-1.62) | 0.84 (0.46-1.55) |
| Q4 | 11,578 | 34 | 126,747 | 0.27 | 1.17 (0.69-1.98) | 1.17 (0.63-2.14) | 1.15 (0.69-1.93) | 1.15 (0.65-2.06) |
| *P*_trend_ |  |  |  |  | 0.736 | 0.875 | 0.750 | 0.970 |

**Supplementary Table 2.** **Association between BMI variability and incident prostate, lung, colon and ovarian cancers based on sensitivity analysis.**

|  |  |  |  |  | **Excluding participants whose PLCO cancers were**  **ascertained in the first two years** | |
| --- | --- | --- | --- | --- | --- | --- |
| **Outcomes** | **N** | **Cases** | **Person-years** | **IR^*^** | **Model 1** | **Model 2** |
| **Lung cancer** |  | 736 |  |  |  |  |
| **SD** |  |  |  |  |  |  |
| Q1 | 22,426 | 164 | 249,132 | 0.66 | 1.00 (reference) | 1.00 (reference) |
| Q2 | 22,437 | 194 | 247,381 | 0.78 | 1.33 (1.08-1.64) | 1.29 (0.99-1.67) |
| Q3 | 22,457 | 202 | 246,481 | 0.82 | 1.57 (1.26-1.95) | 1.48 (1.13-1.93) |
| Q4 | 22,446 | 176 | 244,447 | 0.72 | 1.74 (1.35-2.24) | 1.53 (1.12-2.08) |
| *P*_trend_ |  |  |  |  | **<0.001** | **0.004** |
| **CV** |  |  |  |  |  |  |
| Q1 | 22,442 | 158 | 248,995 | 0.63 | 1.00 (reference) | 1.00 (reference) |
| Q2 | 22,441 | 197 | 247,519 | 0.80 | 1.37 (1.11-1.70) | 1.32 (1.02-1.72) |

**Supplementary Table 2. continued**

|  |  |  |  |  | **Excluding participants whose PLCO cancers were**  **ascertained in the first two years** | |
| --- | --- | --- | --- | --- | --- | --- |
| **Outcomes** | **N** | **Cases** | **Person-years** | **IR^*^** | **Model 1** | **Model 2** |
| Q3 | 22,441 | 200 | 246,268 | 0.81 | 1.56 (1.26-1.95) | 1.51 (1.16-1.97) |
| Q4 | 22,442 | 181 | 244,658 | 0.74 | 1.68 (1.32-2.14) | 1.44 (1.07-1.93) |
| *P*_trend_ |  |  |  |  | **<0.001** | **0.008** |
| **VIM** |  |  |  |  |  |  |
| Q1 | 22,442 | 149 | 248,958 | 0.60 | 1.00 (reference) | 1.00 (reference) |
| Q2 | 22,441 | 193 | 246,856 | 0.78 | 1.43 (1.15-1.78) | 1.41 (1.08-1.83) |
| Q3 | 22,443 | 182 | 246,485 | 0.74 | 1.46 (1.17-1.83) | 1.37 (1.04-1.80) |
| Q4 | 22,440 | 212 | 245,141 | 0.86 | 1.91 (1.53-2.39) | 1.73 (1.32-2.28) |
| *P*_trend_ |  |  |  |  | **<0.001** | **<0.001** |
| **ARV** |  |  |  |  |  |  |
| Q1 | 22,547 | 152 | 250,940 | 0.61 | 1.00 (reference) | 1.00 (reference) |
| Q2 | 22,401 | 195 | 247,365 | 0.79 | 1.49 (1.20-1.86) | 1.50 (1.15-1.96) |

**Supplementary Table 2. continued**

|  |  |  |  |  | **Excluding participants whose PLCO cancers were**  **ascertained in the first two years** | |
| --- | --- | --- | --- | --- | --- | --- |
| **Outcomes** | **N** | **Cases** | **Person-years** | **IR^*^** | **Model 1** | **Model 2** |
| Q3 | 22,351 | 198 | 245,112 | 0.81 | 1.79 (1.43-2.24) | 1.77 (1.34-2.33) |
| Q4 | 22,467 | 191 | 244,024 | 0.78 | 2.15 (1.68-2.76) | 2.17 (1.60-2.94) |
| *P*_trend_ |  |  |  |  | **<0.001** | **<0.001** |
| **Colon cancer** |  | 767 |  |  |  |  |
| **SD** |  |  |  |  |  |  |
| Q1 | 22,381 | 153 | 249,106 | 0.61 | 1.00 (reference) | 1.00 (reference) |
| Q2 | 22,391 | 182 | 247,357 | 0.74 | 1.15 (0.92-1.43) | 1.06 (0.81-1.37) |
| Q3 | 22,424 | 227 | 246,572 | 0.92 | 1.42 (1.14-1.77) | 1.33 (1.02-1.72) |
| Q4 | 22,399 | 205 | 244,308 | 0.84 | 1.36 (1.06-1.75) | 1.30 (0.96-1.76) |
| *P*_trend_ |  |  |  |  | **0.004** | **0.032** |
| **CV** |  |  |  |  |  |  |
| Q1 | 22,399 | 154 | 248,987 | 0.62 | 1.00 (reference) | 1.00 (reference) |

**Supplementary Table 2. continued**

|  |  |  |  |  | **Excluding participants whose PLCO cancers were**  **ascertained in the first two years** | |
| --- | --- | --- | --- | --- | --- | --- |
| **Outcomes** | **N** | **Cases** | **Person-years** | **IR^*^** | **Model 1** | **Model 2** |
| Q2 | 22,400 | 187 | 247,576 | 0.75 | 1.19 (0.96-1.48) | 1.19 (0.92-1.54) |
| Q3 | 22,393 | 228 | 246,160 | 0.92 | 1.44 (1.16-1.79) | 1.38 (1.07-1.79) |
| Q4 | 22,403 | 198 | 244,620 | 0.80 | 1.32 (1.04-1.67) | 1.30 (0.97-1.73) |
| *P*_trend_ |  |  |  |  | **0.001** | **0.041** |
| **VIM** |  |  |  |  |  |  |
| Q1 | 22,399 | 158 | 248,971 | 0.63 | 1.00 (reference) | 1.00 (reference) |
| Q2 | 22,399 | 211 | 246,909 | 0.85 | 1.35 (1.09-1.66) | 1.35 (1.05-1.72) |
| Q3 | 22,398 | 192 | 246,423 | 0.78 | 1.23 (0.99-1.53) | 1.12 (0.86-1.45) |
| Q4 | 22,399 | 206 | 245,040 | 0.84 | 1.36 (1.09-1.70) | 1.37 (1.05-1.79) |
| *P*_trend_ |  |  |  |  | **0.027** | 0.089 |
| **ARV** |  |  |  |  |  |  |
| Q1 | 22,497 | 144 | 250,909 | 0.57 | 1.00 (reference) | 1.00 (reference) |

**Supplementary Table 2. Continued**

|  |  |  |  |  | **Excluding participants whose PLCO cancers were**  **ascertained in the first two years** | |
| --- | --- | --- | --- | --- | --- | --- |
| **Outcomes** | **N** | **Cases** | **Person-years** | **IR^*^** | **Model 1** | **Model 2** |
| Q2 | 22,368 | 190 | 247,348 | 0.77 | 1.37 (1.10-1.71) | 1.32 (1.02-1.71) |
| Q3 | 22,358 | 217 | 245,785 | 0.88 | 1.58 (1.26-1.98) | 1.51 (1.15-1.97) |
| Q4 | 22,372 | 216 | 243,302 | 0.89 | 1.67 (1.30-2.14) | 1.56 (1.15-2.11) |
| *P*_trend_ |  |  |  |  | **<0.001** | **0.003** |
| **Prostate cancer** |  | 4,138 |  |  |  |  |
| **SD** |  |  |  |  |  |  |
| Q1 | 10,659 | 1,051 | 119,538 | 8.79 | 1.00 (reference) | 1.00 (reference) |
| Q2 | 10,659 | 1,075 | 118,511 | 9.07 | 1.05 (0.97-1.15) | 1.04 (0.94-1.15) |
| Q3 | 10,659 | 1,071 | 118,003 | 9.08 | 1.08 (0.99-1.18) | 1.08 (0.98-1.20) |
| Q4 | 10,659 | 941 | 117,306 | 8.02 | 1.00 (0.91-1.11) | 1.00 (0.89-1.13) |
| *P*_trend_ |  |  |  |  | 0.75 | 0.69 |
| **CV** |  |  |  |  |  |  |

**Supplementary Table 2. Continued**

|  |  |  |  |  | **Excluding participants whose PLCO cancers were**  **ascertained in the first two years** | |
| --- | --- | --- | --- | --- | --- | --- |
| **Outcomes** | **N** | **Cases** | **Person-years** | **IR^*^** | **Model 1** | **Model 2** |
| Q1 | 10,659 | 1,033 | 119,611 | 8.64 | 1.00 (reference) | 1.00 (reference) |
| Q2 | 10,659 | 1,070 | 118,484 | 9.03 | 1.06 (0.97-1.16) | 1.03 (0.93-1.13) |
| Q3 | 10,659 | 1,073 | 117,793 | 9.11 | 1.09 (1.00-1.20) | 1.07 (0.96-1.19) |
| Q4 | 10,659 | 962 | 117,470 | 8.19 | 1.02 (0.93-1.12) | 1.01 (0.91-1.13) |
| *P*_trend_ |  |  |  |  | 0.55 | 0.62 |
| **VIM** |  |  |  |  |  |  |
| Q1 | 10,659 | 1,028 | 249,141 | 8.61 | 1.00 (reference) | 1.00 (reference) |
| Q2 | 10,659 | 1,060 | 247,395 | 8.94 | 1.05 (0.96-1.14) | 1.01 (0.92-1.12) |
| Q3 | 10,659 | 1,046 | 246,546 | 8.86 | 1.06 (0.97-1.15) | 1.03 (0.93-1.14) |
| Q4 | 10,659 | 1,004 | 244,408 | 8.56 | 1.02 (0.94-1.12) | 1.02 (0.92-1.13) |
| *P*_trend_ |  |  |  |  | 0.59 | 0.66 |
| **ARV** |  |  |  |  |  |  |

**Supplementary Table 2. continued**

|  |  |  |  |  | **Excluding participants whose PLCO cancers were**  **ascertained in the first two years** | |
| --- | --- | --- | --- | --- | --- | --- |
| **Outcomes** | **N** | **Cases** | **Person-years** | **IR^*^** | **Model 1** | **Model 2** |
| Q1 | 10,656 | 1,079 | 119,754 | 9.01 | 1.00 (reference) | 1.00 (reference) |
| Q2 | 10,678 | 1,105 | 118,862 | 9.30 | 1.05 (0.97-1.15) | 1.07 (0.97-1.19) |
| Q3 | 10,640 | 1,051 | 117,759 | 8.93 | 1.03 (0.94-1.13) | 1.04 (0.93-1.15) |
| Q4 | 10,662 | 903 | 116,984 | 7.72 | 0.93 (0.85-1.03) | 0.97 (0.86-1.09) |
| *P*_trend_ |  |  |  |  | 0.21 | 0.54 |
| **Ovarian cancer** |  | 118 |  |  |  |  |
| **SD** |  |  |  |  |  |  |
| Q1 | 11,572 | 34 | 129,773 | 0.26 | 1.00 (reference) | 1.00 (reference) |
| Q2 | 11,577 | 26 | 129,061 | 0.20 | 0.82 (0.48-1.38) | 0.65 (0.36-1.19) |
| Q3 | 11,577 | 29 | 127,846 | 0.23 | 0.96 (0.55-1.67) | 0.74 (0.39-1.42) |
| Q4 | 11,572 | 29 | 126,635 | 0.23 | 1.04 (0.53-2.01) | 0.91 (0.42-1.98) |
| *P*_trend_ |  |  |  |  | 0.87 | 0.72 |

**Supplementary Table 2. Continued**

|  |  |  |  |  | **Excluding participants whose PLCO cancers were**  **ascertained in the first two years** | |
| --- | --- | --- | --- | --- | --- | --- |
| **Outcomes** | **N** | **Cases** | **Person-years** | **IR^*^** | **Model 1** | **Model 2** |
| **CV** |  |  |  |  |  |  |
| Q1 | 11,575 | 32 | 129,715 | 0.25 | 1.00 (reference) | 1.00 (reference) |
| Q2 | 11,574 | 29 | 128,873 | 0.23 | 0.97 (0.58-1.61) | 0.85 (0.47-1.52) |
| Q3 | 11,574 | 28 | 127,827 | 0.22 | 0.97 (0.56-1.69) | 0.86 (0.46-1.62) |
| Q4 | 11,575 | 29 | 126,899 | 0.23 | 1.07 (0.58-1.99) | 0.95 (0.46-1.94) |
| *P*_trend_ |  |  |  |  | 0.85 | 0.85 |
| **VIM** |  |  |  |  |  |  |
| Q1 | 11,575 | 29 | 129,562 | 0.22 | 1.00 (reference) | 1.00 (reference) |
| Q2 | 11,574 | 34 | 128,827 | 0.26 | 1.25 (0.75-2.07) | 1.18 (0.67-2.08) |
| Q3 | 11,574 | 25 | 128,181 | 0.19 | 0.95 (0.54-1.66) | 0.76 (0.39-1.48) |
| Q4 | 11,575 | 30 | 126,744 | 0.24 | 1.19 (0.68-2.08) | 1.13 (0.59-2.14) |
| *P*_trend_ |  |  |  |  | 0.79 | 0.96 |

**Supplementary Table 2. Continued**

|  |  |  |  |  | **Excluding participants whose PLCO cancers were**  **ascertained in the first two years** | |
| --- | --- | --- | --- | --- | --- | --- |
| **Outcomes** | **N** | **Cases** | **Person-years** | **IR^*^** | **Model 1** | **Model 2** |
| **ARV** |  |  |  |  |  |  |
| Q1 | 11,553 | 34 | 129,818 | 0.26 | 1.00 (reference) | 1.00 (reference) |
| Q2 | 11,762 | 21 | 131,268 | 0.16 | 0.66 (0.38-1.15) | 0.63 (0.33-1.18) |
| Q3 | 11,310 | 35 | 124,862 | 0.28 | 1.24 (0.73-2.12) | 1.04 (0.55-1.94) |
| Q4 | 11,673 | 28 | 127,367 | 0.22 | 1.06 (0.56-2.03) | 1.17 (0.56-2.46) |
| *P*_trend_ |  |  |  |  | 0.49 | 0.55 |

**Supplementary Table 3. Association between repeated BMI measurement and incident prostate, lung, colon and ovarian cancers.**

|  | Model 1 | | Model 2 | |
| --- | --- | --- | --- | --- |
| Outcome | HR (95%CI) | *P* value | HR (95%CI) | *P* value |
| Lung cancer | 0.98 (0.96-1.00) | 0.050 | 0.99 (0.97-1.01) | 0.329 |
| Colon cancer | 1.01 (0.99-1.03) | 0.067 | 1.02 (0.99-1.04) | 0.054 |
| Prostate cancer | 0.99 (0.98-1.00) | 0.333 | 0.99 (0.98-1.00) | 0.129 |
| Ovarian cancer | 0.99 (0.95-1.03) | 0.476 | 0.98 (0.93-1.02) | 0.310 |

Model 1 was adjusted for age, sex and BMI at baseline. Model 2 of lung cancer was adjusted for age, sex, education, BMI at baseline, race, smoking and drinking status, randomization arm, vegetable and fruit consumption, family history of lung cancer, vitamin D intake, physical activity and the cross-product term of physical activity and fruit intake. Model 2 of colon cancer was adjusted for age, sex, education, BMI at baseline, race, smoking and drinking status, randomization arm, vegetable and fruit consumption, family history of colon cancer, vitamin D intake, physical activity and colon comorbidities. Model 2 of prostate cancer was adjusted for age, education, BMI at baseline, race, smoking and drinking status, randomization arm, vegetable and fruit consumption, family history of prostate cancer, vitamin D intake and physical activity. Model 2 of ovarian cancer was adjusted for age, education, BMI at baseline, race, smoking and drinking status, randomization arm, vegetable and fruit consumption, family history of ovarian cancer, vitamin D intake and physical activity.
