# Supplementary figures and images for: Variability of body mass index and risks of prostate, lung, colon, and ovarian cancers
Source: Front Public Health. 2022 Aug 25;10:937877. doi: 10.3389/fpubh.2022.937877 (PMC9452651; doi:10.3389/fpubh.2022.937877)

Figure S1

(A) Age

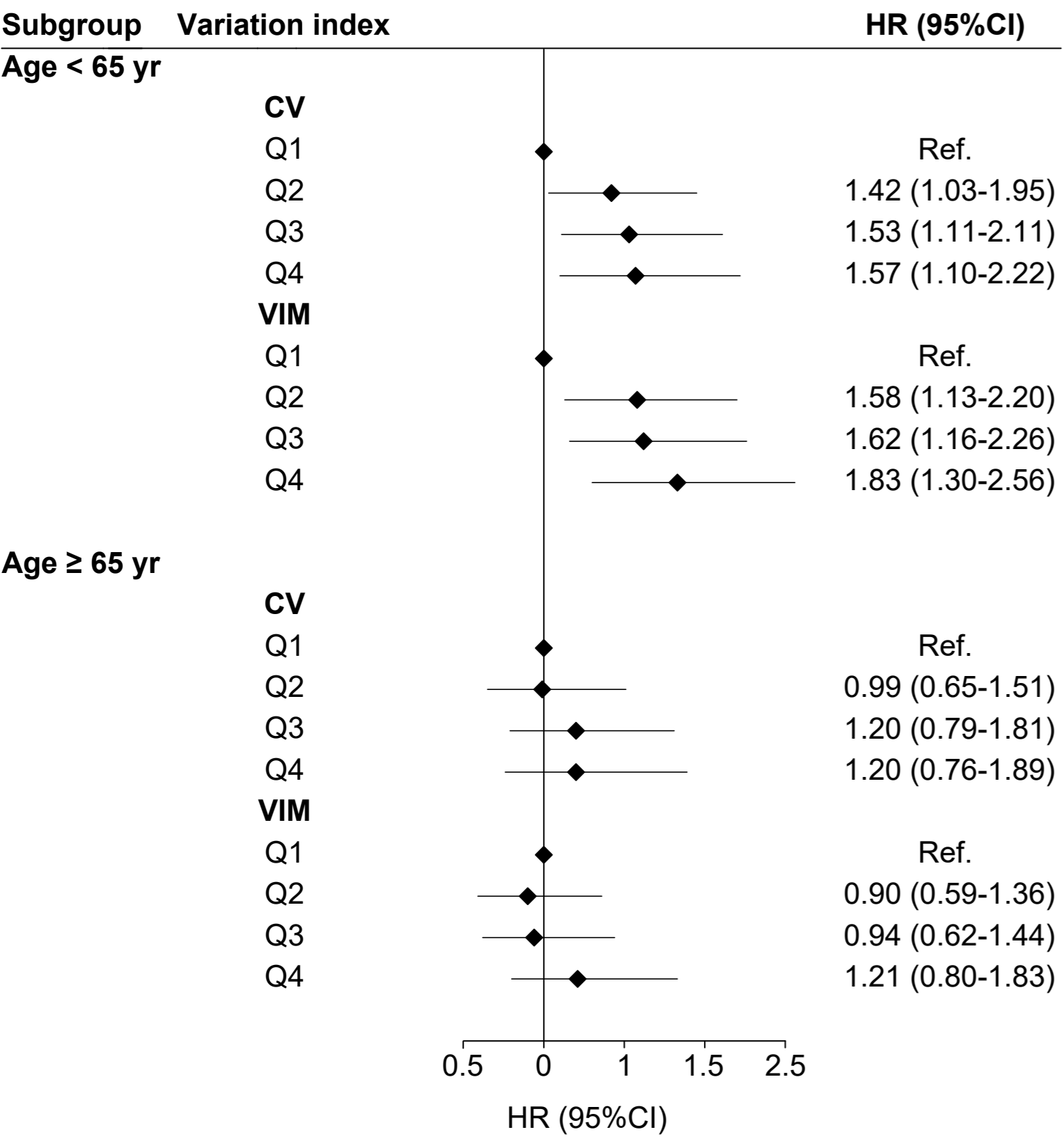

(B) Sex

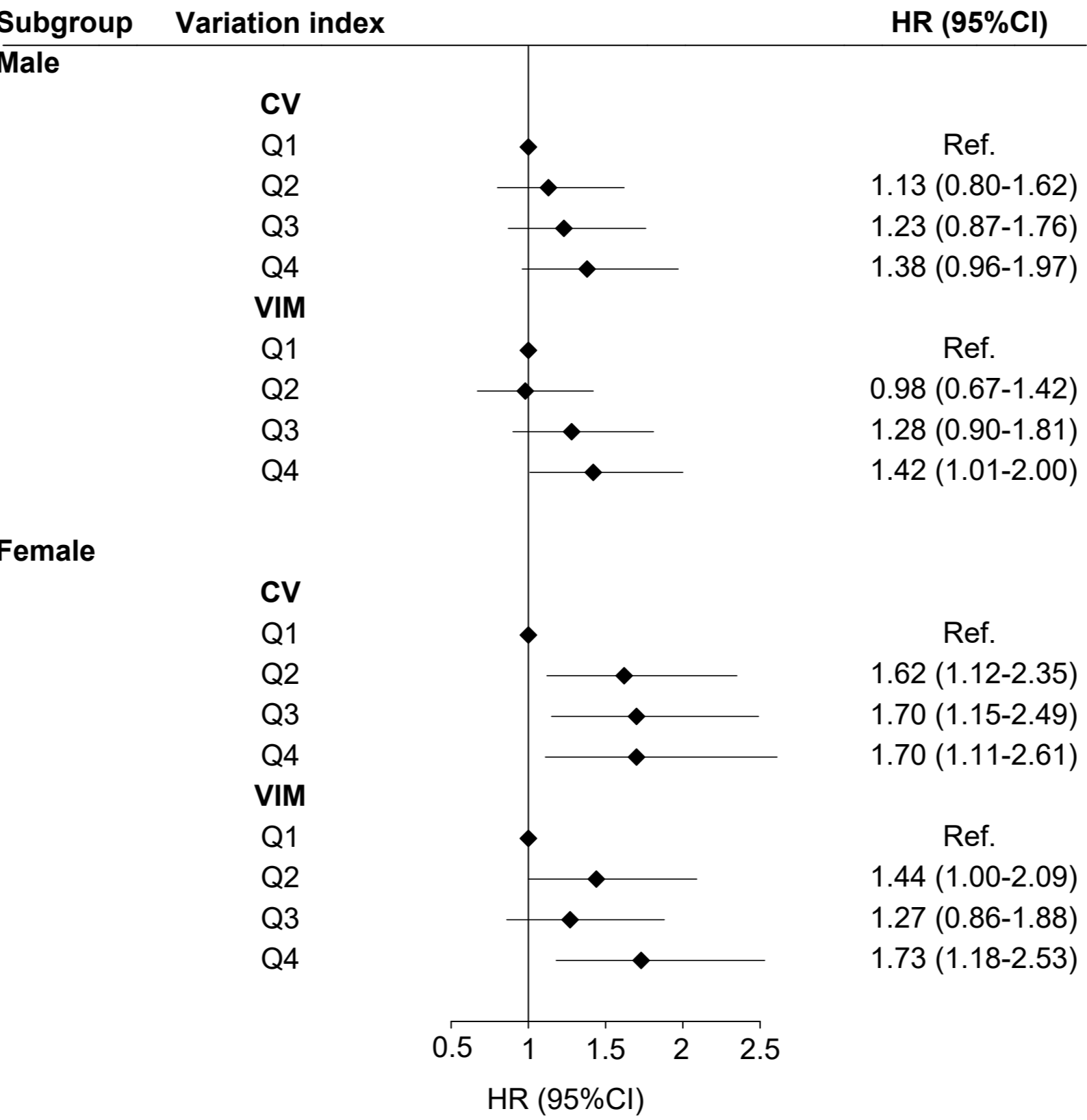

(C) Smoking status

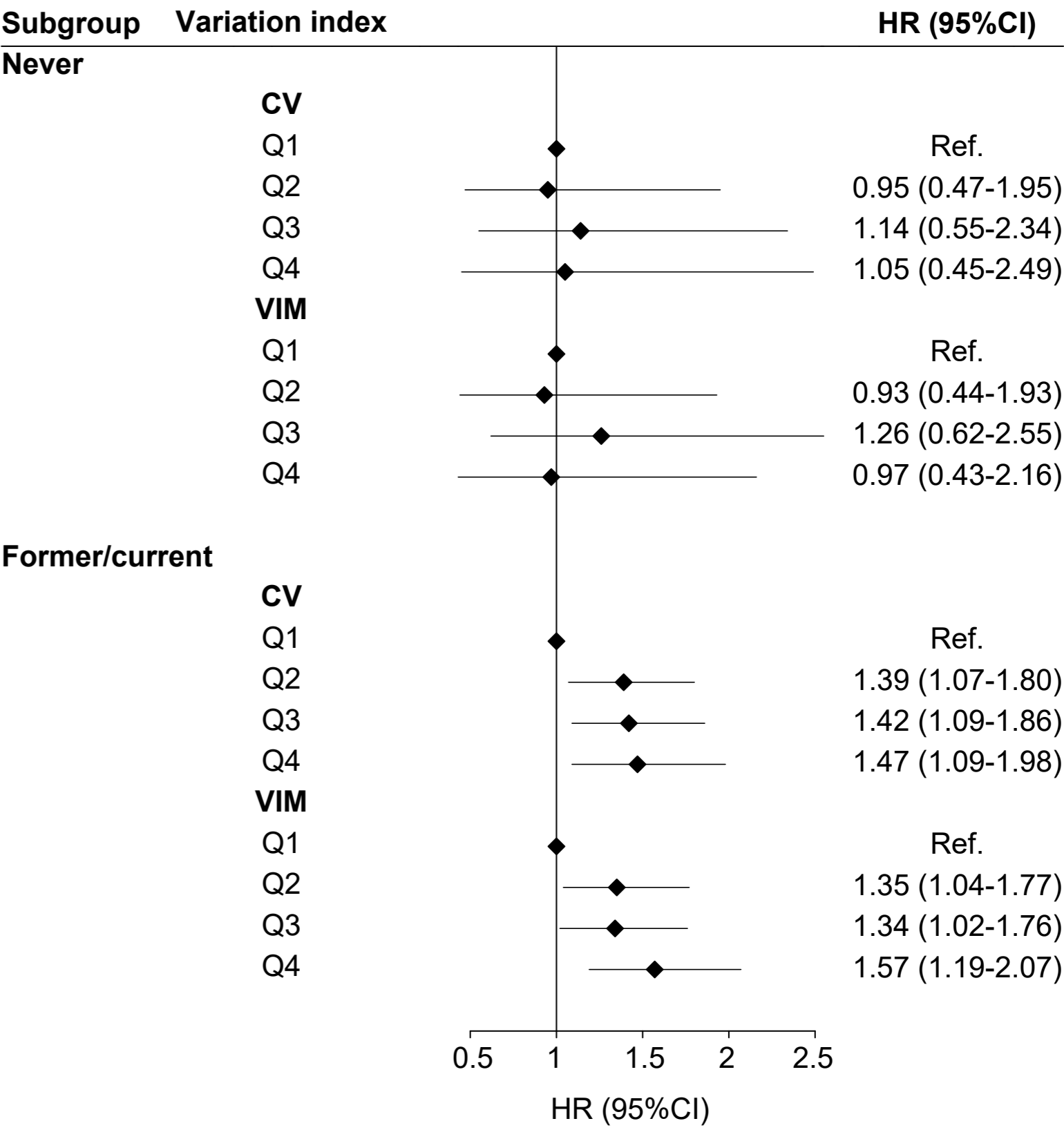

(D) BMI trajectory

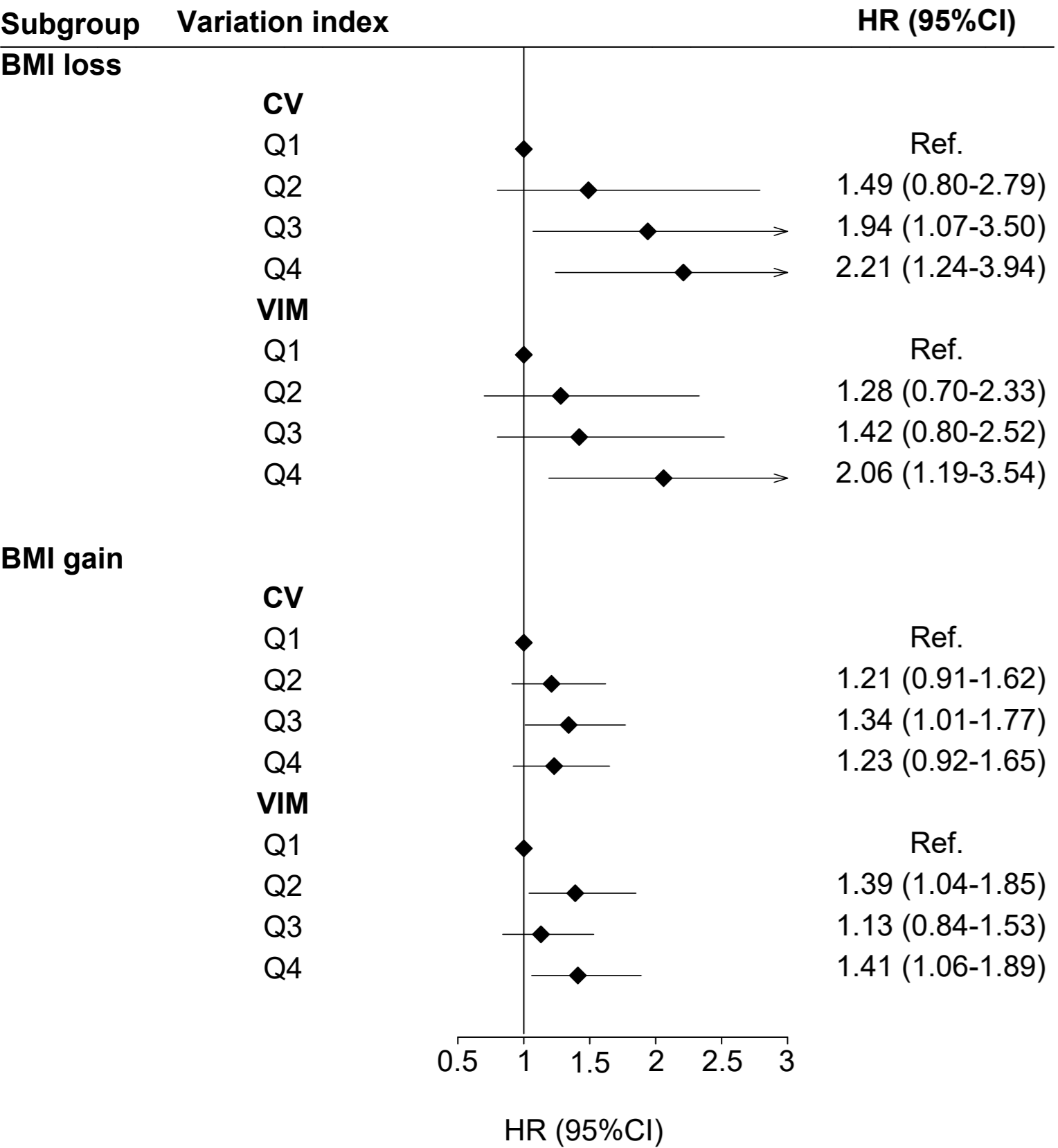

(E) Baseline BMI

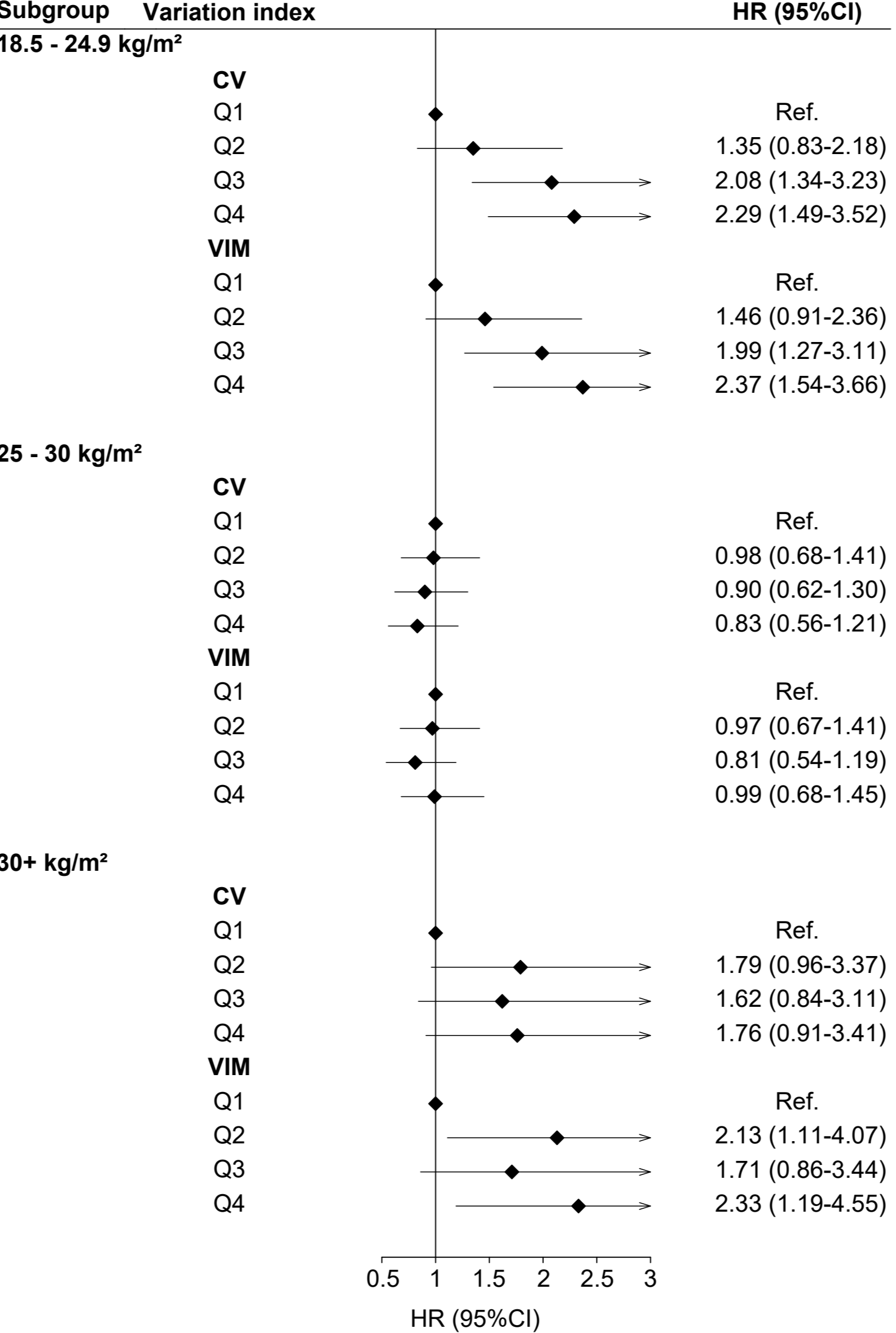

Supplement: Supplementary file 1 [file Data_Sheet_1.ZIP › Supplementary Material/Supplementary Figure 1.pdf]

Figure S2

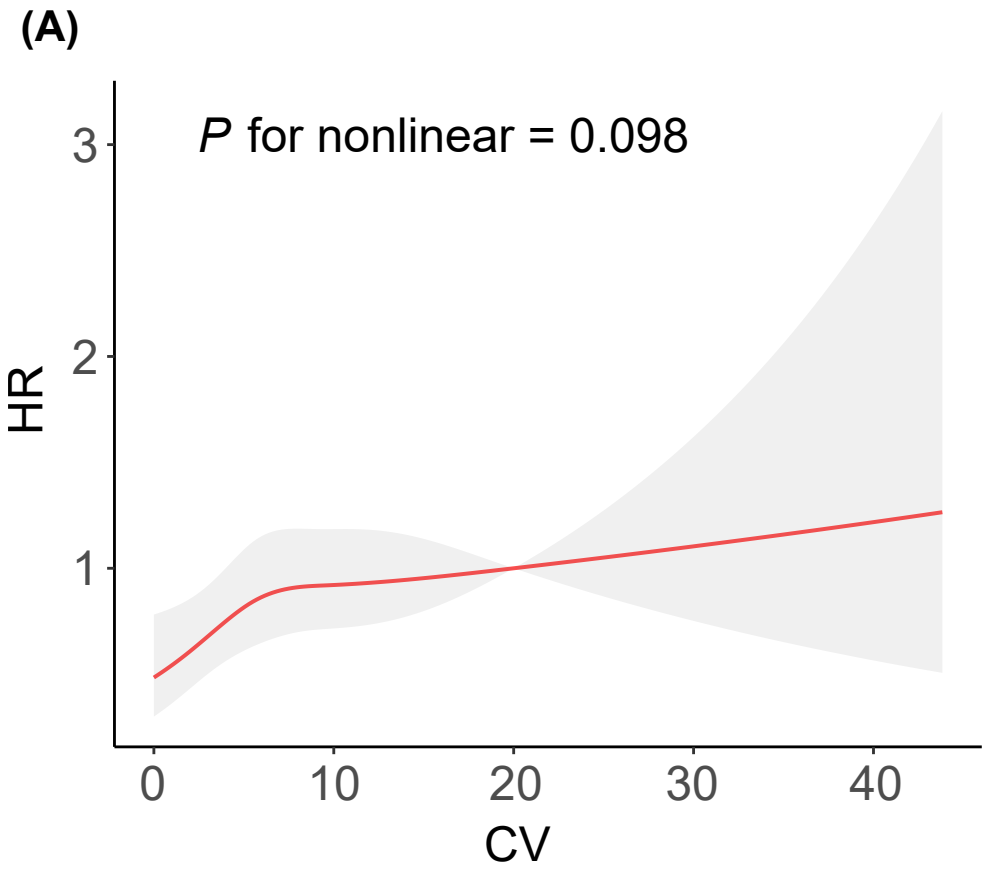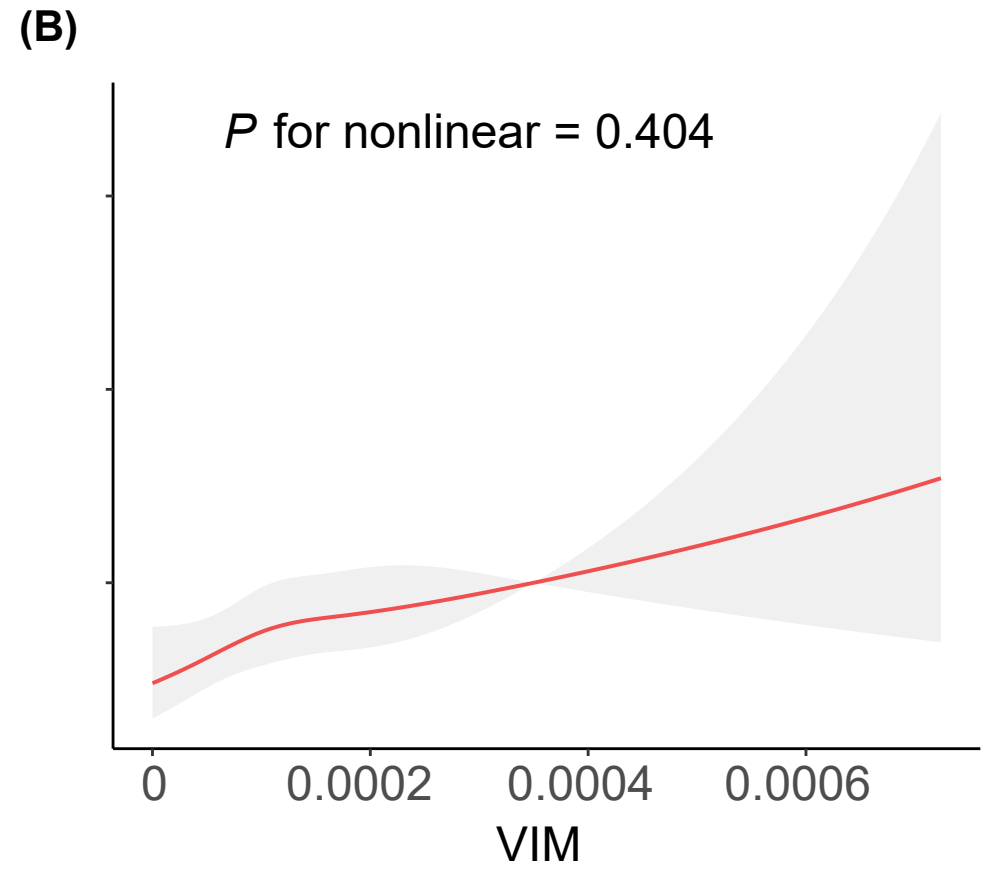

Supplement: Supplementary file 1 [file Data_Sheet_1.ZIP › Supplementary Material/Supplementary Figure 2.pdf]
